# Influence of the Mineral Powder Content on the Asphalt Aging Resistance in High-Altitude Areas Based on Indoor Ultraviolet Light Tests

**DOI:** 10.3390/ma13030754

**Published:** 2020-02-06

**Authors:** Xiangbing Xie, Huixia Li, Junchao Duan, Guanghui Li, Shenjia Tong

**Affiliations:** 1 School of Civil Engineering and Architecture, Zhengzhou University of Aeronautics, Zhengzhou 450046, Henan, China; lgh@163.com; 2 School of Civil Engineering, Fujian University of Technology, Fuzhou 350118, Fujian, China; 19792120@fjut.edu.cn; 3 Huazhong University of Science and Technology, Wuhan 430074, Hubei, China; flyboy1129@163.com; 4 CCTEB Infrastructure Construction Investment CO., LTD, Wuhan 430074, Hubei, China; 5 School of Civil Engineering, Xi’an University of Architecture and Technology, Xi’an 710055, Shanxi, China; tongshenjia@163.com

**Keywords:** asphalt mortar: UV aging, physical properties, rheological properties, standard residual square sum method, high-altitude regions

## Abstract

Intense ultraviolet irradiation is an important environmental factor affecting the service performance of asphalt mixtures in high-altitude areas, and the asphalt mortar is the main factor affecting the durability of asphalt mixtures. It is of great theoretical significance and engineering value to study the performance of the asphalt mortar at medium and low temperatures under ultraviolet irradiation. Therefore, this paper focuses on the evolution of the effect of the filler content on the rheological properties of different asphalt materials at low and medium temperatures under quantitative UV irradiation. Taking the average amount of UV irradiation observed annually in Northwest China as the indoor aging condition, the matrix asphalt mortar and modified asphalt mortar with different mass ratios of asphalt mortar are selected for indoor aging tests. Physical property tests, low-temperature performance tests, and dynamic shear rheological tests are carried out. The effects of the UV irradiation intensity and mineral powder content on the low temperature performance of the asphalt mortar are studied by variance analysis method, and the reasonable mass ratio range of the asphalt mortar under UV irradiation is proposed based on the standard residual square sum (STRSS) method. The results show that the temperature sensibility and low-temperature deformation energy significantly decrease with the increase in the filler content, while the values of the softening point, fatigue factor (G*sin *δ*), and creep stiffness modulus of the asphalt mortar increase. In addition, the variance analysis of the creep stiffness modulus aging index (*SAI*) shows that the ultraviolet radiation intensity has a significant impact on the performance of the asphalt mortar. When the mineral powder content is less than 40%. When the filler content is greater than 40%, the filler content effects the performance of the asphalt mortar. According to the standard residual square sum (STRSS) method, the best mass ratio of the base asphalt mortar is 1.096, and the best mass ratio of the modified asphalt mortar is 0.9091.

## 1. Introduction

Intense ultraviolet irradiation is one of the common environmental and climatic characteristics in high-altitude areas [1]. Therefore, it is of great theoretical significance to study the service performance and life of asphalt pavement under ultraviolet irradiation. The asphalt mortar plays a role in binding the aggregates and filling the voids in asphalt mixtures, and it determines the mechanical properties and durability of the mixture [2,3]. If the amount of mineral powder is low, it will not be enough to absorb free asphalt to form ‘structural asphalt’, and too much mineral powder will cause the asphalt mastic to agglomerate, which will cause asphalt pavement segregation and adverse consequences [4]. Therefore, it is of great significance to study the UV aging resistance characteristics of the asphalt mortar and to determine the appropriate amount of mineral powder based on UV aging resistance in high-altitude areas.

The reproduction of indoor UV irradiation conditions is the key condition to determine the aging rule of the asphalt mastic. However, at the present time, there are no relevant design codes or standards for the aging conditions or for the selection of the UV light source, irradiation intensity, or ambient temperature. Henglong Zhang et al. and Jiangying Yu et al. simulated the UV aging process in a draft oven with a 500 W UV lamp at 80 °C [5,6]. Fen Ye et al. conducted UV aging in a UV box with a 3.08 W lamp at 40 °C [7]. Virginie Mouillet et al. researched the different thickness of asphalt film on aging in a UV chamber with 0.44 W/m^2^ fluorescent lamps at 60 °C [8]. Naskar stored 2 mm thick bitumen films in a UV chamber maintained at room temperature for 30 min with a 500 W UV lamp [9]. Panfeng Du et al. investigated UV aging using a draft oven with a 500 W UV lamp at 60 °C [10]. Feipeng Xiao et al. conducted UV aging in a box with 8000 µW/cm^2^ UVA intensity at 80 °C [11]. Yue Xiao et al. studied asphalt binders using TFOT aging in ultraviolet radiation chambers at 50 °C using 35 W/m2 ultraviolet lamps with a radiation intensity of 35 W/m^2^ [12]. Zhengang Feng et al. placed 2 mm thick asphalt films into an UV irradiation oven with a 500 W UV lamp, and the average intensity of the UV irradiation on the bitumen surface was approximately 800 μW/cm^2^ [13]. Some other studies that include different UV aging conditions are shown in Table 1. Researchers are interested in UV intensity, temperature, and light source selection for UV aging tests.

From the perspective of composite materials, an asphalt mixture is composed of an asphalt mortar with viscoelastic properties and aggregates that form the skeletal structure [2]. The aging resistance of asphalt mixtures mainly depends on the asphalt mortar, which is composed of asphalt, mineral powder, and an interfacial phase formed in the contact zone between the two [21,22]. Similar to asphalt materials, many researchers think that the aging processes of asphalt mortar also mainly include thermal oxidative aging, photo-oxidative aging, etc. The aging of the asphalt mortar results in an increase in the stiffness modulus and a decrease in the adhesiveness [23,24,25]. However, researchers have conducted extensive research on the thermal aging of asphalt mortar. For example, Recasens studied the influence of fillers on the anti-aging performance of asphalt by analyzing the changes in penetration, softening points, and viscosity indices of asphalt mortar under different aging times and found that the optimum content of fillers was 20%–30% [26]. Wang et al. studied the high and low temperature performance of asphalt mortar at different aging stages using the rolling thin film oven tests and found that the mineral powder had a great influence on the mortar aging performance when the mass ratio of the mineral powder to mortar was less than 1.5 and that increasing the mineral powder content can lower the rate of asphalt aging [27]. Cheng et al. used the thin film oven test to study the effect of diatomite and mineral powder on the thermal oxidative aging properties of asphalt and concluded that the aging of asphalt was reduced by diatomite and mineral powder, which suggested that the optimal content of diatomite for engineering was 12.8%. In addition, the anti-aging effect of diatomite was better than that of mineral powder as a result of its porous structure [28]. Liu Guoqiang’s study showed that the low temperature properties of asphalt mortar were worse with increases in the aging degree [29]. Huang Shinche combined different modelling techniques to investigate the effect of fillers on the long-term aging characteristics of asphalt binders and found that a generalized power law model could be used to characterize the asphalt binders, in which the stiffness effect of asphalt filler was reduced with aging [30]. However, Xie investigated the anti-ultraviolet aging mechanism of asphalt mortar from the perspective of physical chemistry by infrared spectroscopy and found that the degree of degradation of the SBS modifier increased from slight to severe with increasing amounts of mineral powder [14]. Additionally, some work has been done to examine the role of fillers in the aging process of asphalt mortar. For example, Cheng et al. studied the influence of mineral powder on thermal oxidative aging properties of asphalt and concluded that mineral powder prevented the interconnection of internal components of asphalt so that the effect of aging on the thermal susceptibility of asphalt could be weakened [28]. Moraes and Bahia investigated effects of the mineral fillers on stiffness and glass transition temperature of PAV-aged asphalt binder. The results showed that a selected filler concentration and mineralogy type might reduce the oxidative aging of asphalt binder [31]. Zhang et al. analyzed the effects of various material properties of filler, including filler particle size, SiO_2_ content on asphalt-filler interaction ability, and found that the relative proportion between ’free asphalt’ and ’structural asphalt’ was significantly influenced by filler particle size [32]. Wang researched the anti-aging mechanism of diatomite modified asphalt mortar and concluded that the diatomite unique micropore structure hindered the asphalt oxidative aging [33]. Zhao Lin studied the microstructure of the different mass ratio of asphalt mortar under oxidative aging conditions by atomic force microscopy (AFM). The results showed that the addition of mineral powder would hinder the diffusion of oxygen molecules, slowing the aging of matrix asphalt [34]. Naveed et al. studied that the viscosity–temperature relationship of the amount of fillers effect on the asphalt mortar, and found that the micro-bearing effect of filler would facilitate the compaction of asphalt mixture [35]. Xie Xiangbing et al. was studied the effects of the amount of mineral powder in asphalt mortar on the ultraviolet aging properties of asphalt were investigated by Fourier transform infrared spectrometry (FTIR) and gel permeation chromatography (GPC). The result showed that, with the addition of the filler, the index of butadiene double bonds and the values of peak broadening of the modifier phase firstly increased and then decreased. This might be explained as follows: with an increasing amount of mineral powder, the oil content in the bitumen begins to decrease, to reduce the swelling effect of the SBS modifier and to lead the SBS modifier to increase the degree of degradation in the UV radiations [14].

In conclusion, the research on the thermal oxidative aging of asphalt mortar has achieved fruitful results, but the research on the photo oxidative aging of asphalt mortar is not perfect, especially with respect to the aging resistance of asphalt materials with the varying amounts of mineral powder. Therefore, this paper first analyzes UV aging conditions indoors. On this basis, a homemade environmental UV aging box is used to study the physical performance, low temperature performance and rheological properties of matrix asphalt mortar and modified asphalt mortar before and after aging under different mass ratios of mineral powder to asphalt. In addition, the factors that affect the UV aging resistance of asphalt mortar are analyzed by variance analysis on the basis of low-temperature performance. Finally, the standard residual square sum (STRSS) index method is used to determine the best mass ratio of the asphalt mortar based on UV aging at high altitudes.

## 2. Experimental

### 2.1. Materials

The base asphalt, SK-90, and its SBS-modified asphalt were used in this investigation. The basic physical properties of these two kinds of asphalt are summarized in Table 2. The mineral powder was sieved through a 0.075 mm screen, its specific surface area was 3.834 m^2^/g, and its average grain diameter was 6.510 μm. The particle size distribution is presented in Table 3.

### 2.2. Ultraviolet Aging Method and Preparation of Mastics 

**Figure 1:**
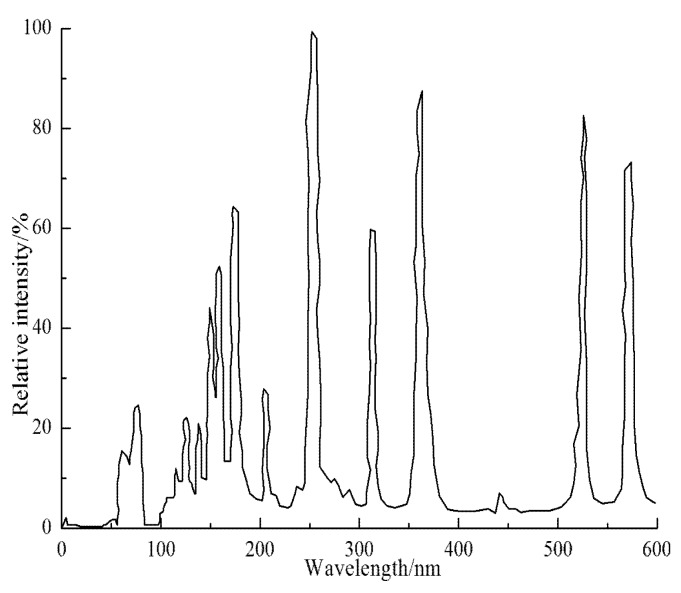
Light spectrum.

In this paper, the environmental UV aging box was produced to meet the experimental needs. This equipment mainly consists of environmental chambers, temperature controls and light sources. The related research results show that the chemical bonds are most sensitive in the wavelength range of 295–365 nm [36,37]. Compared with the spectral distribution of other artificial simulated UV light sources, the main spectral peak of a high-pressure mercury lamp is 365 nm. In addition, this lamp radiates at the wavelengths of 404.7 nm, 435.8 nm, 546.1 nm, and 577.0–579.0 nm [4,13,20,38]. Therefore, the high-pressure mercury lamp was selected as the UV irradiation light source in this paper. The main technical parameters of the UV lamp are shown in Table 4, and its spectrum distribution is shown in Figure 1. In addition, the ultraviolet radiation intensity on the surface of the sample is closely related to the height of the lamp in the box. When the lamp is too close to the test sample, thermal oxidative aging occurs due to the high heat of the lamp. When the lamp is too far away, the ultraviolet radiation intensity decreases, and the indoor ultraviolet radiation time is prolonged. Therefore, this paper determined that the distance between the lamp and the sample surface should be 40 cm. The UV irradiation intensity at the sample surface was measured by a special UV meter, and the UV irradiation intensity was 260 W/m^2^. The indoor UV aging time was determined according to the principle that the indoor and outdoor UV radiation amounts are equal. That is, indoor UV aging time should equal the total amount of natural UV radiation. Therefore, the indoor UV radiation time intensity was set to the annual average amount of UV radiation observed in high-altitude areas, which is 420 MJ/m^2^ [1,3,38,39]. To ensure the service life of the relevant equipment in the environmental UV aging box, it was shut down for 20 min every 8.0 h during the tests. It was calculated that the aging time of the asphalt mortar in the environmental UV box for 1.0 h is equivalent to 19.4 h of outdoor natural UV radiation. In this paper, 194 h was selected as the UV radiation time.

To ensure the uniform UV irradiation of the sample, a rotatable disc holder was set up in the environmental box. The center of rotary table was suspended in the center of the oven on a vertical axis. The driving mechanism rotated the table horizontally at a speed of 5.5 r/min. The inner diameter of the disc is 360 mm. There are four shallow grooves in this disc for the placement of the sample dishes. Each sample dish is made of a silica gel material with an inner diameter of 140 mm and a thickness of 5 mm. A high-pressure mercury lamp generates substantial heat when operating. To prevent the sample from undergoing thermal oxidative aging in addition to ultraviolet irradiation, ventilation devices were used. A 200 W blower was used as the air supply device to provide enough cold air to the box, and it is installed on the left side of high pressure mercury lamp to provide cold air. In addition, a 100 W blower continuously sent the hot air out of the box. The temperature of the environmental box was controlled at 35 °C by the temperature control system described above.

**Figure 2:**
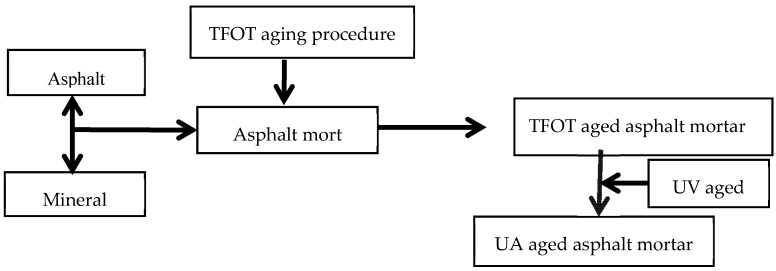
Preparation chart of asphalt mastic.

According to the relevant research results [14,27,29,40], the mass ratios of the mineral powder, or filler, to the base asphalt (F/A) or SBS modified asphalt as were selected as 0, 0.8, 1.0, 1.2, 1.4, and 1.6. The preparation process of asphalt mortar is shown in Figure 2. To ensure the uniform dispersion of the filler in the asphalt, the mineral powder was added to the asphalt in a certain proportion and blended at 1000 r/min. By controlling the total mass of the sample, it was possible to ensure that the film thickness was approximately 2 mm. After all of the samples using the thin film oven test (TFOT) according to the China test specification JTG E20-2011 [41], they were allowed to cool to room temperature and placed in the UV environmental oven. 

### 2.3. Physical Properties Test

The physical properties, including penetration (at 25 °C), softening point and ductility, were analyzed according to ASTM D5, ASTM D36, and ASTM D113, respectively [42,43,44]. The ductility of the asphalt mortar has a good correlation with the test temperature [45,46]. In this paper, the force-ductility test conditions are analyzed at 10 °C and 1 cm/min. The rotational viscosity of the base asphalt mortar and SBS modified asphalt mortar were measured using a Brookfield viscometer at 120 °C, 135 °C and 145 °C in accordance with ASTM D4402 [47]. According to the Arrhenius equation (Equation (1)), the viscous activation energy values (△*E_η_*) of the asphalt mortar before and after aging were calculated under the different mass ratios of mineral powder to asphalt [48]:

(1) $$\ln\eta =\ln A+\frac{\Delta E\eta }{RT}$$

where △*E_η_* is the viscous flow activation energy, kJ/mol; *η* is the apparent viscosity, Pa·s; ***T*** is the absolute thermodynamic temperature, K; *R* is the Boltzmann constant, whose value is 8.314 J/(mol·K); and *A* is an empirical constant [40,48].

Combining the results of the force-ductility test, penetration test and Brookfield viscosity test before and after the aging of the asphalt mortar, the effects of the mineral powder content on the physical properties of matrix asphalt and SBS modified asphalt were evaluated by using the deformation energy aging index (*DEAI*), residual penetration ratio (*RP*), visibility aging index (*VAI*), and activation energy aging index (*EAI*). The values of the *RP*, *DEAI*, *VAI*, and *EAI* were calculated according to Equations (2)–(5), respectively:

(2) $$RP=\frac{Pentration(aged)}{P\mathrm{en}tration(unaged)}\times 100$$

(3) $$DEAI=\frac{Aged\;\mathrm{ductility}\;\mathrm{value}-Unaged\;\mathrm{ductility}\;\mathrm{value}}{Unaged\;\mathrm{ductility}\;\mathrm{value}\;}\times 100$$

(4) $$VAI=lg[\mathrm{lg}(1000\eta_{aged})]-\mathrm{lg}[\mathrm{lg}(1000\eta_{unaged})]$$

(5) $$EAI=\frac{E_{a,aged}-E_{a,unaged}}{E_{a,unaged}}\times 100.$$

### 2.4. DSR and BBR Test

Strong ultraviolet radiation and low temperatures are the typical environmental climate characteristics in high-altitude areas. The fatigue factor (G*sin *δ*) and low-temperature anti-cracking performance of asphalt mortar in asphalt mixture are the main indices affecting the performance of asphalt pavement in high-altitude areas [14,21,41,49]. Therefore, the DSR and BBR tests were conducted to evaluate the rheological properties of the base asphalt mortar and SBS modified asphalt mortar at middle and low temperatures. According to AASHTO T315-05 [50], the DSR test should be conducted at 10 °C and 25 °C, using a fixed speed (10 rad/s). According to AASHTO T313-12 [51], the BBR test should be conducted at −12 °C and −18 °C.

In this paper, the fatigue factor (G*sin *δ*), low temperature creep stiffness modulus (S) and creep stiffness slope (m)indices of the SHRP plan are used to study the changes in the effect of the mineral powder content on the aging resistance of asphalt. The influence of mineral powder content on the UV aging resistance of asphalt was evaluated by calculating the fatigue factor index (*FAI*) and low temperature creep stiffness modulus index (*SAI*) of asphalt mortar before and after aging according Equations (6) and (7), respectively:

(6) $$\;FAI\;=\frac{{\left(G^{*}\cdot \sin\delta \right)}_{aged}-{\left(G^{*}\cdot \sin\delta \right)}_{unaged}}{{\left(G^{*}\cdot \sin\delta \right)}_{unaged}}\times 100.$$

(7) $$SAI=\frac{S_{aged}-S_{unaged}}{S_{unaged}}\times 100.$$

## 3. Analysis of Testing Results 

### 3.1. Physical Properties of the Asphalt Mortar before and after UV Aging

#### 3.1.1. Changes in the Softening Point before and after UV Aging

**Figure 3:**
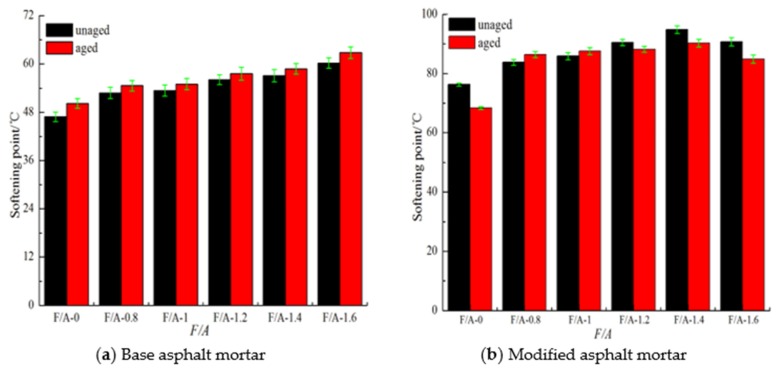
Softening point of asphalt mortar before and after UV aging.

The changes of the softening point of the asphalt mortar before and after UV aging are shown in Figure 3. It can be seen in Figure 3 that, before UV aging, with the increase in the ratio value (filler to asphalt, F/A), the softening points of the two types of asphalt mortar gradually increases, which shows that adding mineral powder can effectively improve the high-temperature stability of asphalt, which is consistent with the conclusion [28,38,52]. Compared with the results before aging, the softening points of the two types of asphalts show different trends after UV aging. The softening point of the base asphalt mortar shows a rising trend after UV aging, while the modified asphalt mortar shows a complex change pattern, that is, the softening point both rises and falls. According to the related result of the SBS modified asphalt after UV aging [32,38], the change trend of the softening point depends on the joint action of the base asphalt and the SBS polymer. The base asphalt UV aging will increase the softening point of the modified asphalt, while the degradation of the SBS polymer will reduce the softening point of the modified asphalt. According to Figure 3b, in the UV aging process of the SBS modified asphalt mortar, when the F/A ratios are 0.8 and 1.0, the aging of the matrix asphalt is the dominant factor, while when the F/A ratios are 0, 1.2, 1.4, and 1.6, the degradation of the SBS polymer is the dominant factor. According to the change in the softening point of the two types of asphalt mortar before and after UV aging, the change in the softening point of the asphalt mortar before and after aging exhibits obvious randomness, especially for the modified asphalt mortar. Therefore, it is not easy to use the change in the softening point to evaluate the influence of the mineral powder content on the anti-UV aging performance of asphalt.

#### 3.1.2. Changes of the Penetration before and after UV Aging at 25 °C

**Figure 4:**
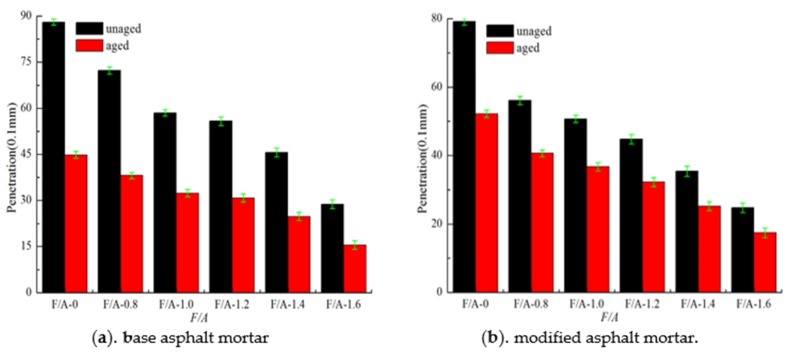
Penetration of asphalt mortar before and after UV aging.

**Figure 5:**
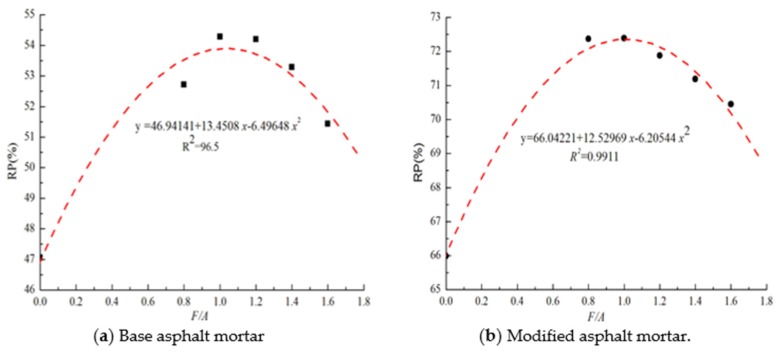
Residue penetration of asphalt mortar.

The penetration values of the two types of asphalt mortar before and after UV aging are shown in Figure 4. Before UV aging, the penetration values of the two types of asphalt mortar decreased gradually, and the penetration value of the modified asphalt mortar was smaller than that of the matrix asphalt mortar. Combined with the softening point values shown in Figure 3, the penetration index (PI) of the modified asphalt mortar was larger than that of the matrix asphalt mortar, which indicates that the temperature sensitivity of the modified asphalt mortar was better than that of the matrix asphalt mortar. Compared with the values before UV aging, the penetration values of the two types of asphalt mortar significantly decreased after aging, and the penetration value of the modified asphalt mortar was greater than that of the base asphalt mortar. The residual penetration ratios (*RP*) of the two types of asphalt mortar are calculated by Equation (2) as shown in Figure 5. It can be seen in Figure 5 that, with the increase in the F/A ratio, the value of *RP* first increase and then decrease, indicating that the addition of mineral powder can effectively improve the light aging resistance of asphalt. Using Figure 5, it can be determined that the value of the best ratio of the mineral powder to the asphalt of matrix asphalt mortar is 1.035, and the value of the best ratio of modified asphalt mortar is 1.010.

#### 3.1.3. Results of the Force-Ductility Experiment before and after UV Aging 

The results of the force-ductility test using the two types of asphalt mortar before and after UV aging are shown in Table 5. It can be seen in Table 5 that before UV aging, when the F/A ratio is 0, the peak force of the matrix asphalt mortar and the modified asphalt mortar are equal, both of which are 23 KN, indicating that the peak force of the modified asphalt depends on the matrix asphalt, and the SBS modifier mainly affects the toughness and fracture lengths of the modified asphalt [53,54]. Compared with the results before aging, the peak force of the two types of asphalt mortar showed a growth trend, in which the maximum increase in the base asphalt was 70 KN, and the maximum increase in the modified asphalt was 53 KN, which was mainly caused by the ultraviolet aging of the asphalt. However, after adding the mineral powder, the minimum increase in the base asphalt mortar was 60 KN, and the minimum increase in the modified asphalt mortar was 50 KN, which indicated that UV aging hardening and filler stiffening both increase the peak force of the neat base asphalt. Different from the trend of change in the peak force of the asphalt mortar, the maximum ductility of the asphalt mortar decreases gradually after adding the mineral powder before UV aging, which is mainly due to the exponential increase in the viscosity and stiffness of the asphalt mortar with the increase in the mineral powder content [38,46]. The mineral powder particles prevent the viscoelastic deformation of the asphalt and reduce the low-temperature anti-deformation performance of the asphalt during the stretching process. Compared with the results before aging, the ratio of ductility difference of the two types of asphalt mortar first decreased and then increased after UV aging. The maximum reduction in the ductility of the matrix asphalt mortar was 56.86% (F/A-0), the maximum reduction in the modified asphalt mortar was 50.85% (F/A-0), the maximum reduction in matrix asphalt mortar after adding mineral powder was 49.84% (F/A-1.4), and the maximum reduction in modified asphalt mortar was 42.24% (F/A-1.2), which shows that adding mineral powder can effectively resist the ultraviolet aging of asphalt.

**Figure 6:**
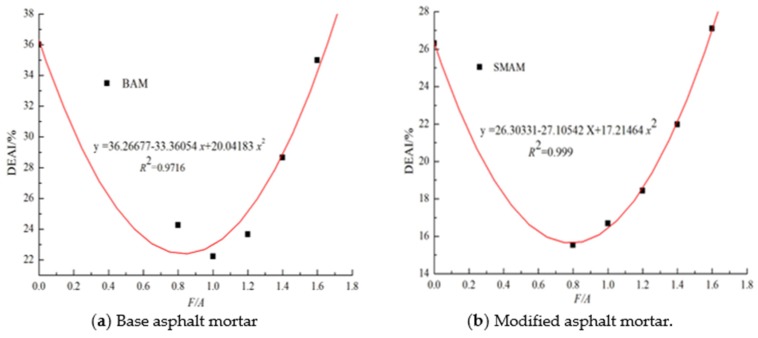
The *DEAI* value of asphalt mortar.

In conclusion, the effect of the mineral powder on the peak force and ductility of asphalt shows different trends, which suggests that the above two evaluation indices cannot effectively evaluate the effect of the mineral powder content on the anti-aging performance of asphalt in UV conditions. Therefore, in this paper, the force-displacement curve of asphalt mortar is integrated to calculate the deformation energy. The results are shown in Table 5. The deformation energy aging index (*DEAI*) is introduced to evaluate the effect of the mineral powder content on the anti-aging performance of the asphalt under UV light. The results are shown in Figure 6. It can be seen that with the increase in the F/A ratio, the *DEAI* first decreases and then increases, and the *DEAI* of the modified asphalt mortar is smaller than that of the matrix asphalt mortar. The higher the deformation energy aging index is, the more serious the aging degree is [28,38], which shows that the addition of an appropriate amount of mineral powder can effectively improve the UV aging and low temperature resistance of the matrix asphalt and modified asphalt. Furthermore, when the F/A ratio is 0.8–1.0, the two types of asphalt mortar show different trends. There may be reason that the filler in the modifier mastic has a different influence on the SBS modifier and base asphalt. This may be explained as follows: with the same amount of mineral powder for the base asphalt mortar and SBS-modified asphalt mortar, the oil content in the bitumen begins to decrease, to reduce the swelling effect of the SBS modifier and to lead the SBS modifier to increase the degree of degradation in the UV radiations [14].

#### 3.1.4. Changes of the Thermal Susceptibility before and after UV Aging

**Figure 7:**
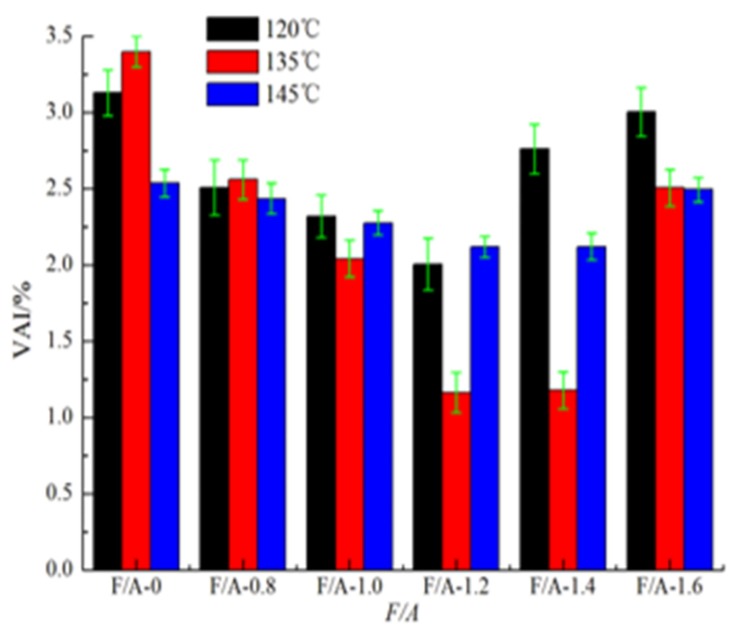
Value of base asphalt mortar.

**Figure 8:**
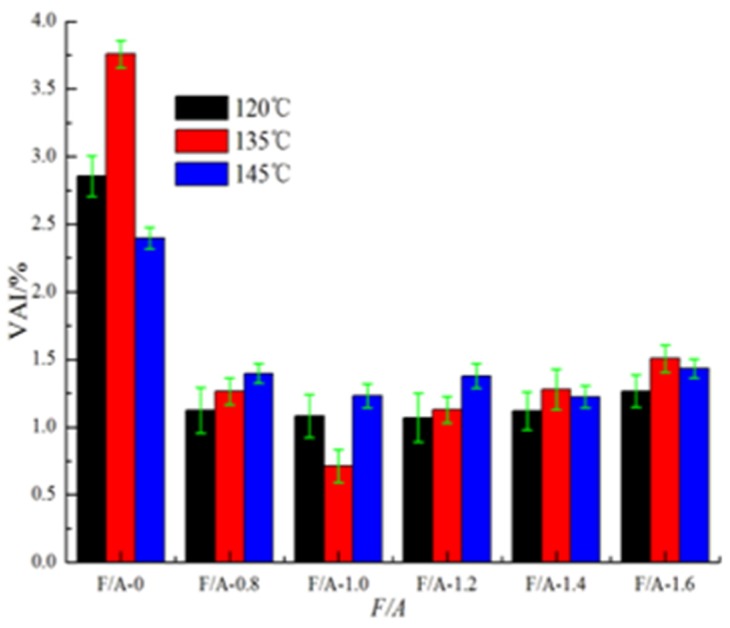
*VAI* value of modified asphalt mortar.

The *VAI* values of the base asphalt mortar and modified asphalt mortar are shown in Figure 7 and Figure 8, respectively. It can be seen in Figure 7 and Figure 8 that at the same temperature, with the increase in the F/A ratio, the *VAI* values of the two types of asphalt mortar show a trend of first decreasing and then increasing, and the *VAI* value of the modified asphalt mortar is lower than that of the base asphalt mortar. Accordingly, the higher the *VAI* value is, the more serious the degree of aging is [28,52]. It can be seen that the UV degree of aging of the base asphalt mortar is greater than that of the modified asphalt mortar and that mineral powder can effectively improve the anti-ultraviolet aging properties of asphalt. At different temperatures, the difference of the *VAI* value of the two types of asphalt mortar with the same ratios of mineral powder to asphalt show different obviously. For the base asphalt mortar, the *VAI* value at 120 °C is the most significant, while for the modified asphalt mortar, the *VAI* value at 135 °C is the most significant.

**Figure 9:**
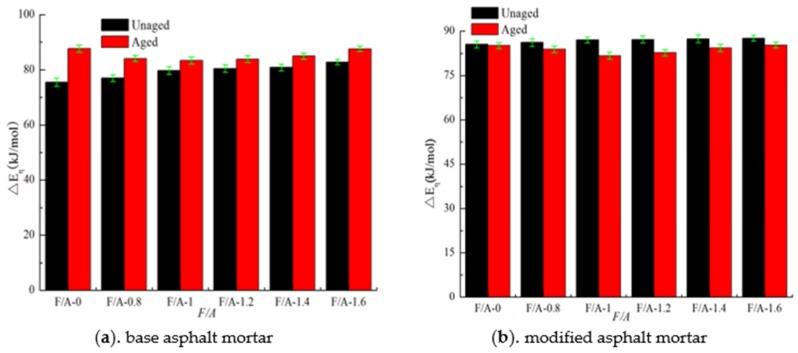
Activation energy about asphalt mortar before and after UV aging.

According to Equation (1), the viscous-flow activation energy (△*E_η_*) before and after UV aging of the asphalt mortar are calculated, and the relevant calculation results are shown in Figure 9. It can be seen in Figure 9 that before UV aging, the viscous-flow activation energy of the two types of asphalt mortar increases with the increase in the mineral powder content. Compared with the results before UV aging, the △*E_η_* value of the matrix asphalt mortar increases, the △*E_η_* value of modified asphalt mortar decreases, and the △*E_η_* value of the modified asphalt mortar is lower than that of the matrix asphalt mortar. The △*E_η_* is closely related to the temperature stability of a material. The higher the viscosity activation energy is, the lower the temperature sensitivity is [22,28,52]. It can be seen that the temperature sensitivity of the matrix asphalt mortar is reduced by UV irradiation, which is mainly due to the interaction between the asphalt components. Compared with the matrix asphalt, the viscosity activation energy of the modified asphalt decreased after UV aging. This result may occur due to the degradation of the SBS under UV irradiation, which weakens the interaction between the asphalt and the polymer [36,38,55].

**Figure 10:**
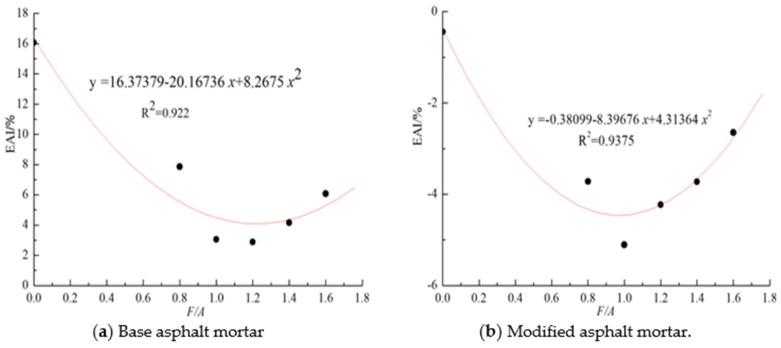
Activation energy index about these different types of asphalt mortar.

According to the *VAI* values of the asphalt mortar at different temperatures, the optimal content of the mineral powder in asphalt mortar cannot be effectively determined. Therefore, by defining the activation energy aging index (*EAI*), this paper analyzes the influence of the mineral powder content on the UV aging resistance of the two types of asphalt. The calculation results are shown in Figure 10. It can be seen in Figure 10a that the *EAI* values of base asphalt mortar are smaller than those of the neat base asphalt. With the increase in the F/A, the *EAI* values of the base asphalt mortar shows a trend of first decreasing and then increasing, which shows that the influence of ultraviolet aging on the activation energy of asphalt mortar is caused by the amount of mineral powder. Adding a proper amount of mineral powder can effectively reduce the effect of ultraviolet aging on the influence of asphalt temperature sensitivity. According to Table 5, with the increase in the activation energy of the asphalt mortar, its low temperature ductility gradually decreases. In addition, as mineral powder is added to improve the anti-ultraviolet aging performance of asphalt, the low temperature performance should be maintained. Therefore, the smaller the *EAI* value of an asphalt mortar is, the better the anti-ultraviolet aging performance of the asphalt is. The results of the above analysis are also applicable to the modified asphalt mortar. According to Figure 10a,b, the value of the best mass ratio of mineral powder to base asphalt mortar is 1.220, while the value of the best ratio of mineral powder to modified asphalt is 0.973.

### 3.2. Changes of the Fatigue Factor (G*sin *δ*) of Asphalt Mortar before and after UV Aging

**Figure 11:**
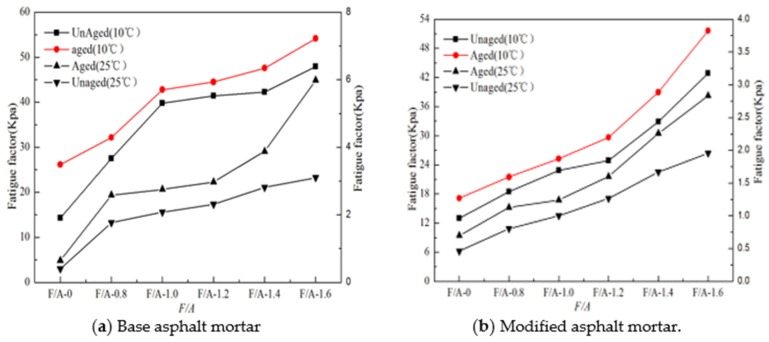
Fatigue factor (G*sin *δ*) on asphalt mortar.

**Figure 12:**
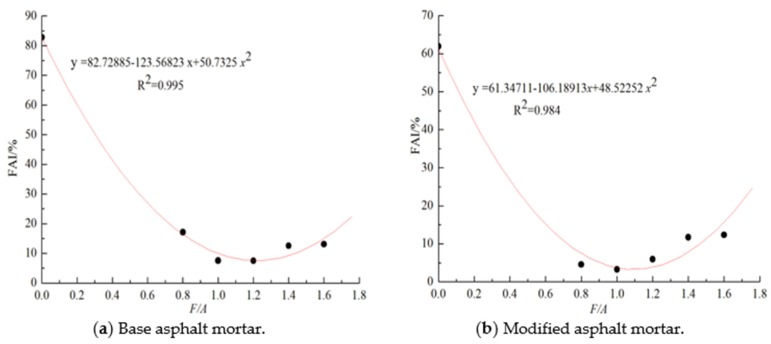
Fatigue factor aging index about these different types of asphalt mortar.

The fatigue factors (G*sin *δ*) values of the matrix asphalt mortar and modified asphalt mortar at 10 °C and 25 °C before and after UV aging are shown in Figure 11a,b, respectively. It can be seen in Figure 11 that the fatigue factor at 10 °C is greater than that at 25 °C under the same ratio of mineral powder to asphalt. Comparing the results of the ratio of mineral powder to asphalt (F/A-0), with the increase in the mineral powder content, the fatigue factors at both temperatures gradually increases, and the influence of the mineral powder content on the fatigue performance of asphalt is smaller than that of the temperature. Compared with the results before aging, at the same mineral powder to binder ratio, the fatigue factor of asphalt mortar after UV aging increases. To further analyze the influence of the mineral powder content on the aging fatigue performance of asphalt, the *FAI* value is calculated using Equation (6), and the results are shown in Figure 12. It can be seen in Figure 12 that compared with neat asphalt, with the increase in the mineral powder content, the *FAI* values of the two types of asphalt mortar decrease, and the *FAI* value of the modified asphalt mortar is smaller than that of matrix asphalt mortar, which shows that the influence of ultraviolet aging on the fatigue performance of asphalt mortar is caused by the mineral powder content, and the addition of an appropriate mineral powder can effectively reduce the effect of ultraviolet aging on the fatigue degradation of asphalt.

### 3.3. Changes of the Low-Temperature Performance of Asphalt Mortar and Analysis of Variance 

#### 3.3.1. Evaluation of the Low-Temperature Performance of Asphalt Mortar

**Figure 13:**
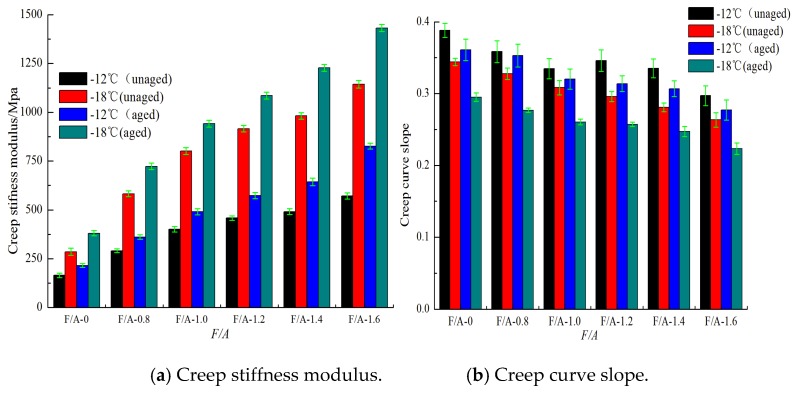
Low temperature performance evaluation parameters of base asphalt mortar.

**Figure 14:**
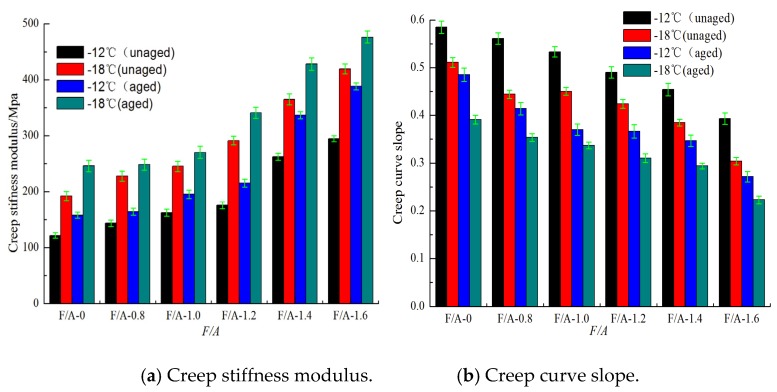
Low-temperature performance evaluation parameters of modified asphalt mortar.

**Figure 15:**
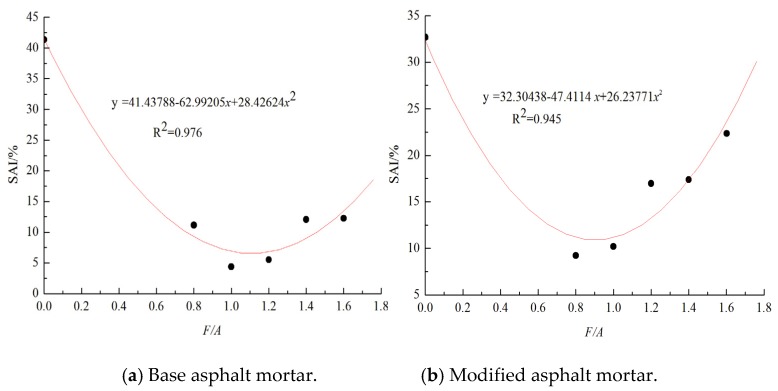
Creep stiffness factor aging index about these different types of asphalt mortar.

Figure 13 and Figure 14 show the creep stiffness modulus and creep curve slope of the base asphalt mortar and modified asphalt mortar before and after UV aging, respectively. It can be seen in Figure 13 and Figure 14 that, under the same ratios of mineral powder to asphalt, as the temperature decreases, the creep stiffness modulus of the asphalt mortar increases, and the creep curve slope decreases. In addition, the lower the temperature is, the more significant the changes in these low temperature performance evaluation indices are. At the same temperature, with the increase in the mass ratio of the mineral powder to asphalt, the creep stiffness modulus value gradually increases, and the creep curve slope decreases. Compared with the results before aging, the creep stiffness modulus increases, and the creep stiffness curve slope decreases at the same ratio. Therefore, the UV aging hardening and mineral powder stiffening both increase the creep stiffness of neat asphalt. The effect of the mineral powder content on the low temperature performance of the asphalt is analyzed by using the low temperature creep stiffness modulus aging index (*SAI*). The results are shown in Figure 15. From Figure 15, it can be concluded that the *SAI* value first decreases and then increases. This result indicates that adding suitable fillers to asphalt can improve its anti-aging properties. The best ratio of the matrix asphalt mortar is approximately 1.108, and that of the modified asphalt mortar is 0.9034.

#### 3.3.2. Analysis of Variance (ANOVA)

**Figure 16:**
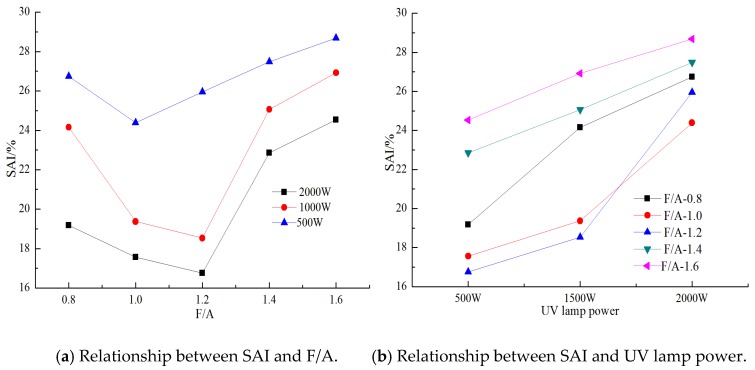
The *SAI* value of asphalt mortar.

To further study the effect of the ultraviolet radiation intensity and mineral powder content on the low temperature anti-cracking performance of the asphalt, the variance analysis method is used to analyze the influence degree of different factors in this paper. Combined with the relationship between the performance of asphalt materials and the climatic characteristics [40,56], this paper evaluates the influence of these two factors on the low temperature anti-cracking performance of the asphalt with the creep stiffness modulus aging index (*SAI*). The test temperature is −18 °C, and the test results are shown in Figure 16a,b, respectively.

It can be seen in Figure 16a that at the same ratio of mineral powder to asphalt, with the increase in the UV lamp power, the *SAI* value gradually decreases, while at larger ratios of mineral powder to asphalt, the effect of the UV lamp power on the *SAI* value decreases. From Figure 16b, it can be seen that under different UV lamp powers, the change curves of *SAI* value of the different ratios of mineral powder to asphalt are parallel. These results show that there is no interaction between the power of UV lamp and the ratio of mineral powder to asphalt [57]. Therefore, the variance of the two influencing factors when the ratio of mineral powder to asphalt is 0.8–1.4 and 1.2–1.6 are studied, respectively. The analysis results are shown in Table 6 and Table 7.

The greater the sum of the mean square deviations of a factor is, the more significant the influence of this factor is [58]. It can be seen that when the F/A ratio is 0.8–1.4, the UV irradiation intensity has a significant impact on the *SAI* value, while when the F/A ratio is 1.2–1.6, the mineral powder content in the F/A ratio has a significant impact on the *SAI* value. Through the conversion of the volume and mass of asphalt mortar, it can be determined that when the volume of the mineral powder is less than 40%, the influence of the ultraviolet irradiation intensity can be considered, and when the volume of mineral powder is more than 40%, the influence of the mineral powder content is the most significant. When studying the performance of the asphalt mortar under ultraviolet irradiation, the effect of the mineral powder content cannot be ignored.

### 3.4. Optimal Content of the Mineral Powder for the Anti-UV Aging Purpose

Through the analysis of the influence of the mineral powder content on the asphalt medium and low temperature performance under UV irradiation, it can be seen that the mineral powder content has a significant impact on the aging resistance of the asphalt. Through the data fitting method, the optimal content of the mineral powder in the asphalt mortar under the UV irradiation based on the rheological properties at medium and low temperatures can be calculated. However, by using a multi-objective optimization algorithm, we can determine the normalized ratio of the mineral powder to asphalt that can meet the requirements of low- and medium-temperature performance while undergoing ultraviolet ageing. That is, we can determine the best mineral powder to asphalt ratio to facilitate engineering applications [28,56]. The change in the *RP* ratio with the F/A ratio exhibits a convex parabola, which is opposite to those of the *DEAI*, *EAI*, *FAI*, and *SAI* indices. This result may be caused by the compound UV-aging hardening and fillers stiffening. Therefore, the *RP* ratio is taken as the control condition, and the other indices are taken as the optimization analysis indices. When the square sum of standard residuals (*STRESS*) result reaches the minimum value, the optimal ratio of mineral powder to asphalt, i.e., *y* (*i, optimal*), is obtained based on the better performance at medium and low temperatures under UV irradiation [59]. The *STRESS* value can be calculated using Equation (8):

(8) $$STRESS=100\sqrt{\frac{\sum_{i=1}^{4}{\left[y(i,j)-y(i,optimal)\right]}^{2}}{\sum_{i=1}^{4}{\left[y(i,optimal)-y(i,0)\right]}^{2}}}$$

where *y* (*i*, *j*) is the value of the *i* index when the ratio of the mineral powder to asphalt is *j*, and *y* (*i*, optimal) is the value of the *i* index when the ratio of the mineral powder to asphalt is optimal. *y* (*i*, 0) is the value of the base asphalt or modified asphalt in this paper. 

The allowable error precision of the control input variable, y, and the target function STRSS is 0.01%. By means of cyclic iteration, the optimal ratio of the mineral powder to asphalt is determined when Equation (8) reaches its minimum value. The calculation results are shown in Table 8 and Table 9. From Table 8 and Table 9, it can be concluded that the different types of asphalt mortar can reach a uniform powder binder ratio by using a multi-objective optimization algorithm. Under this ratio of mineral powder to asphalt, the error between the performance evaluation index values at medium and low temperatures and the ratio of mineral powder to asphalt of a single index are within 5%, which meets the needs of the project. Through the optimization method, the best ratio of mineral powder to modified asphalt is 0.9091, and that of matrix asphalt mortar is 1.0960.

## 4. Conclusions

In this research, investigations on the filler content of different types of asphalt mortar under ultraviolet irradiation was carried out and the content effects on anti-aging performance of asphalt materials were evaluated by the conventional performance tests and rheological tests. The following summary and conclusions can be made:

(1) The conventional performance tests results showed that the addition of mineral powder can increase the softening point, peak force and viscosity while decreasing the penetration of the original bitumen. The DSR results showed that the anti-fatigue performance of asphalt mortar decreases significantly in contrast to the original bitumen and SBS modified bitumen. Considering the effects of the mineral powder content on the anti-aging properties of asphalt, these evaluation indices of *DEAI*, *EAI*, and *FAI* show the trend of first increasing and then decreasing. Thus, adding appropriate fillers to asphalt can effectively improve its anti-aging ability.

(2) Compared with original asphalt, BBR results showed that the low temperature performance of the two types of asphalt mortar significantly decreased with the increase in the mineral powder content. Additionally, the results of variance analysis show that the content of the mineral powder is 40%, the intensity of the ultraviolet radiation is the most significant factor, and when it is more than 40%, the content of the mineral powder is the most significant factor.

(3) The influence of the filler content on the anti-ultraviolet aging of asphalt is significant. The unified optimal ratio of the mineral powder to asphalt for engineering applications can be determined using the standard residuals sum of squares (STRSS) method. The best mass ratio of base asphalt mortar is 1.0960, and the best mass ratio of modified asphalt mortar is 0.9091.

## Figures and Tables

**Table 1:** Aging conditions of previous researches.

Author	Aging Conditions		
	Light Sources	UV Intensity	Temperature
Xiangbing Xie [14]	High pressure mercury lamp (UV)	200 W/m^2^	35 °C
Liping Liu [15]	High pressure mercury lamp (Reflection type black)	3.4 × 10^4^ μW/m^2^	40 °C
Y.T. Wu [16]	High pressure mercury lamp (Reflection type black)	400 W/m^2^	50 °C
Zhaoyi He [17]	High pressure mercury lamp (UV)	40 W/m^2^	70 °C
He Xie [18]	Fluorescent UV lamps	0.89 W/m^2^·nm	45 °C
Mouillet Virginie [19]	Fluorescent UV lamps	0.72 W/m^2^/nm	45 °C
Wenbo Zeng [20]	High pressure mercury lamp (UV)	500 μW/m^2^	30 °C 50 °C 70 °C

**Table 2:** Physical properties of base asphalt and modified asphalt.

Property		SBS Modified Asphalt	SK-90
Density (15 °C)/(g/cm^3^)		1.032	1.035
Penetration (25 °C, 100g, 5s)/(0.1 mm)		73.1	90
Softening point T_R&B_/°C		76	46
Ductility (15 °C, 5 cm/min)/cm		>100	>100
Penetration index (PI)		0.037	–1.37
After RTFOT 163 °C, 85 min	Mass loss/%	–0.11	0.13
	Penetration ratio of 25 °C/%	86	60.0
	Ductility (15 °C, 5 cm/min)/cm	27.5	15.8

**Table 3:** Particle size distribution of mineral filler.

Particle Size /μm	63.42	30.70	14.26	6.76	2.43	1.26
Passing by mass/%	100	91.3	72.2	47.0	21.43	12.37

**Table 4:** Technical parameters of 1000 W high-pressure mercury lamp.

Types	Photoelectric Parameters			Geometric Parameter		Power density/W/cm	Life span/h
	power/W	voltage/V	current/A	length/mm	diameter/mm		
GY-1000	1000	135	8.1	225	25	80	1000

**Table 5:** Results of force-ductility test (10 °C, 1 cm/min).

Type	Property	Unaged						UV aged					
		0	0.8	1.0	1.2	1.4	1.6	0	0.8	1.0	1.2	1.4	1.6
BAM	F_max_(N)	23	36	49	58	72	94	93	97	112	125	132	157
	D(cm)	51	14.3	11.1	6.8	5.9	3.4	22	7.8	6.1	3.9	2.9	2.2
	E(N·cm)	214.6	279.9	255.2	265.6	245.1	222.1	291.8	347.8	311.9	328.4	315.3	299.8
SMAM	F_max_(N)	23	49	58	74	97	126	76	99	125	138	165	178
	D(cm)	79.7	16.9	16.1	11.6	9	6.5	39	9.8	9.5	6.7	5.2	3.8
	E(N·cm)	1124.3	1208.2	486.2	441.5	294.9	240.3	1420	1395.9	567.3	522.9	359.7	305.4

Note: Energy (E) represents the integral of force-displacement curve.

**Table 6:** Analysis for the ratio of asphalt mortar on 0.8–1.4.

Source	df	Mean Square	F	F_a_
Ratio of mineral powder to asphalt	3	16.165	8.403	F_1–0.001_(3, 6) = 9.78
Ultraviolet radiation intensity B	2	50.632	26.318	F_1–0.001_(2, 6) = 10.9
Error	6	1.924		

**Table 7:** Analysis for the ratio of asphalt mortar on 1.2–1.6.

Source	df	Mean Square	F	F_a_
Ratio of mineral powder to asphalt	2	24.838	23.874	F_1–0.001_(2, 4) = 18.0
Ultraviolet radiation intensity B	2	10.379	9.976	F_1–0.001_(2, 4) = 18.0
Error	4	1.040		

**Table 8:** Results of unified optimal F/A of modified asphalt mortar.

Types	Original			After Optimization		
	Evaluation Indices	F/A	Value of Evaluation Indices (%)	F/A	Value of Evaluation Indices (%)	Error (%)
Modified asphalt	*RP*	1.0100	72.3670	0.9091	72.3135	1.4557
	*DEAI*	0.7873	15.6336	0.9091	15.9289	1.8539
	*EAI*	0.9730	−4.4672	0.9091	−4.2958	3.8369
	*FAI*	1.0940	3.2497	0.9091	3.4120	4.7568
	*SAI*	0.9034	10.8863	0.9091	10.9784	0.8460

**Table 9:** Results of unified optimal F/A of base asphalt mortar.

Types	Original			After Optimization		
	Evaluation Indices	F/A	Value of Evaluation Indices (%)	F/A	Value of Evaluation Indices (%)	Error (%)
Base asphalt	*RP*	1.0350	53.9038	1.0960	54.2625	0.6654
	*DEAI*	0.8323	22.3842	1.0960	23.0144	2.8154
	*EAI*	1.2200	4.0750	1.0960	4.2398	3.8867
	*FAI*	1.2170	7.4857	1.0960	7.6768	2.4887
	*SAI*	1.1080	6.5406	1.0960	6.8157	4.2603
